# Factors associated with hepatitis A susceptibility among men who have sex with men using HIV pre-exposure prophylaxis in Northeastern Brazil: A cross-sectional study

**DOI:** 10.1371/journal.pone.0301397

**Published:** 2024-03-28

**Authors:** Hareton Teixeira Vechi, Mônica Baumgardt Bay, Cláudio Henrique Silva de Freitas, Júlia Gomes Fernandes Costa de Sant’anna, Carlos Brites, Kenio Costa de Lima

**Affiliations:** 1 Institute of Tropical Medicine of Rio Grande do Norte, Federal University of Rio Grande do Norte, Natal, Rio Grande do Norte, Brazil; 2 Multicampi School of Medical Sciences, Federal University of Rio Grande do Norte, Caicó, Rio Grande do Norte, Brazil; 3 Department of Infectious Diseases, Federal University of Rio Grande do Norte, Natal, Rio Grande do Norte, Brazil; 4 Hospital Universitário Prof. Edgard Santos, Federal University of Bahia, Salvador, Bahia, Brazil; 5 Department of Odontology, Federal University of Rio Grande do Norte, Natal, Rio Grande do Norte, Brazil; University of New South Wales, AUSTRALIA

## Abstract

Hepatitis A virus (HAV) infection has disproportionately affected more men who have sex with men (MSM), occurring in outbreaks, despite being vaccine-preventable. We determined the prevalence and factors associated with HAV susceptibility among cisgender MSM on HIV pre-exposure prophylaxis (PrEP) in Northeastern Brazil. From September 30, 2021 to June 19, 2023, 282 cisgender MSM receiving HIV PrEP were enrolled into this cross-sectional study. Sociodemographic and clinical information were collected. Blood samples were collected for screening of sexually transmitted infections (STIs) and serum samples were tested for IgM and total anti-HAV antibodies. Non-reactive results for total anti-HAV antibodies were found in 106 of 282 (37.6%) participants. Factors associated with HAV susceptibility included age <30 years (prevalence ratio [PR]: 2.02; 95% confidence interval [95% CI]: 1.61–2.53), having health insurance (PR: 1.39; 95% CI: 1.19–1.64), sex only with cisgender men (PR: 1.52; 95% CI: 1.23–1.89), non-steady partner (PR: 1.20; 95% CI: 1.01–1.43) and no lifetime history of STIs (PR: 1.25; 95% CI: 1.03–1.53). Identifying clinical correlates of HAV susceptibility in key populations is a fundamental step towards development of public policy focused on prevention, especially following the recent hepatitis A outbreak in Brazil.

## Introduction

Hepatitis A is an acute liver infection caused by hepatitis A virus (HAV), a nonenveloped RNA virus, and humans are the only known natural reservoir [1]. HAV is highly contagious and its transmission is faecal-oral via consumption of contaminated food or water or through direct person-to-person contact, including sexual contact [1]. In 2019, there were an estimated 158.94 million new cases of hepatitis A globally, but the mortality rate was low (0.52 deaths per 100,000) [2]. HAV infection has traditionally been considered an early childhood disease, but the epidemiology of infection has changed over time in many regions of the world, because of routine childhood vaccination and the improvement of socioeconomic conditions, resulting in a shift in the susceptible population to HAV toward adolescents and mostly adults [3, 4].

Brazil has a low-intermediate level of HAV endemicity as defined by World Health Organization (WHO), with about 2,000 people affected by HAV per year [4, 5]. Since 2014 there has been a reduction in the incidence of infection in children under 10 years of age while the number of adolescents and adults with hepatitis A has increased, age groups where the disease tends to be symptomatic and may be more severe [6]. In 2017 and 2018, a hepatitis A outbreak occurred in the city of São Paulo, with a 14-fold increase in the incidence rate, affecting mainly men who have sex with men (MSM) [6, 7].

Hepatitis A has disproportionately affected MSM, including people living with HIV infection and persons receiving HIV pre-exposure prophylaxis (PrEP). Over the past three decades, outbreaks occurred among MSM communities across the world [8–12]. Sexual practices and other behaviors (e.g., substance abuse) that enable faecal-oral transmission are associated with the increased number of cases of HAV infection detected in this group [7, 8, 10, 12]. The transmission of HAV during sex may occur through direct oral-anal (as rimming), digital-anal (as fingering and fisting) and genital-oral (anal sex followed by fellatio) contact, or indirect route by sharing sex toys [13]. Therefore, hepatitis A can be considered a sexually transmitted infection (STI) under certain conditions [14, 15].

Among MSM at risk for HIV infection, PrEP services offer a singular opportunity for integration with viral hepatitis care, including hepatitis A, as HIV and HAV share overlapping risk factors for transmission [16]. According to the Brazilian Guidelines for Pre-Exposure Prophylaxis for Risk of HIV Infection, every HIV PrEP user should be evaluated for HAV susceptibility by testing for IgG or total anti-HAV antibodies [17].

Knowledge of HAV immune status is relevant in making individual decisions about recommending vaccination and providing guidance on preventive measures for susceptible people. In Brazil, the hepatitis A vaccine is only available for children <5 years old or people older than 1 year with chronic risk condition for severe illness [7]. MSM are not included in the Brazilian immunization program. The HAV seroprevalence among MSM varies from 37.0% in Amsterdam to 62.3% in Central Brazil [18, 19]. However, population immunity levels > 70% are needed to prevent outbreaks among MSM [20].

Understanding the epidemiology of HAV infection in PrEP-using MSM can contribute to implementation of targeted preventive public policies. There is an evidence gap in the published literature about HAV seroprevalence among MSM, a potential source for hepatitis A outbreaks. In the present work we sought to determine the prevalence of HAV susceptible individuals and the factors associated with HAV susceptibility.

## Materials and methods

This cross-sectional study was carried out at the Institute of Tropical Medicine of Rio Grande do Norte (IMT-RN), in Northeastern Brazil. The institute is a medical assistance and clinical research unit of Federal University of Rio Grande do Norte (UFRN) and the main public HIV PrEP service, serving 81.1% (585) of all PrEP users in the State of Rio Grande do Norte [21]. The study was approved by the Ethics Committee on Human Research of UFRN, under the protocol number—CAAE:31650520.0.2005.5292. Informed consent about the study was obtained by a written, signed and dated informed consent form from all participants.

The inclusion criteria were cisgender MSM ≥18 years old who started HIV PrEP or were already taking it on the day of the interview. Cisgender MSM were defined as males whose current gender identity is the same as the sex they were assigned at birth and engage in sexual relationships with other males. The term “MSM” was used clinically to refer to sexual behavior alone, regardless of self-described sexual orientation [22]. People with HIV infection, defined according to the diagnostic criteria established by the Brazilian Ministry of Health, were not eligible to enter the study [23]. Although potentially included in the MSM classification due to their sex assignment at birth, transgender women were also excluded, because they represented only 2.8% of patients followed up at IMT-RN [21].

The recruitment of participants was undertaken between September 30, 2021 and June,19 2023 by convenience sampling as participants attended scheduled PrEP visits at IMT-RN. The sample size was calculated using the website OpenEpi (https://www.openepi.com/SampleSize/SSPropor.htm), based on the prevalence of 62.3% for total anti-HAV antibodies among MSM [19], confidence limits of **±** 6%, 5% of α-error and 20% of β-error. The study should include at least 251 individuals.

The participants underwent an interview with a standardized questionnaire to collect sociodemographic characteristics (age, race/skin color, marital status, sexual orientation, education, income and health insurance) and clinical information: hepatitis A and B vaccination status, sexual practices (condomless anal intercourse, sexual role during anal intercourse, frequency of condom use, number and gender of partners, steady partner, exchange sex for money and goods, sex worker, oral sex, sexualized drug use), behaviors potentially associated with oral-anal contact (gay sauna attendance, sharing insertive sex toys, fisting, fingering, rimming, group sex), other behaviors (binge drinking, substance use, erectile dysfunction drug use, geosocial dating app use, length of HIV PrEP use) and medical history of STIs.

Condomless anal sex was defined as penile-anal intercourse without condom. Oral sex was considered penile-oral intercourse. Sexualized drug use consisted of intentional drug use before or during sex to facilitate, enhance, prolong and sustain sexual activity. Sharing insertive sex toys was considered as use of sex toys that are inserted into the anus. Fisting consisted of inserting fist into rectum. Fingering and rimming were defined as using finger and tongue to provide stimulation to the anus, respectively. Group sex was defined as having sex with more than one individual at the same time.

The hepatitis A and B vaccination status was based on the self-report of receipt of, at least, one dose of hepatitis A vaccine and the complete hepatitis B vaccine schedule (three-dose series). We considered binge drinking as consuming ≥5 drinks of alcohol in 2 hours. Substance use was defined as use of any of the following: marijuana, cocaine, poppers, solvents and club drugs (amphetamines, hallucinogens, LSD, ketamine, GHB, ecstasy, bath salts). Erectile dysfunction drug use consisted of taking any drug of phosphodiesterase inhibitors class (e.g., sildenafil). We considered the use of the following dating apps: Grindr^®^, Hornet^®^, Scruff^®^, Tinder^®^, Badoo^®^ and Growlr^®^. A medical history of STIs comprised any of the following: syphilis, anogenital wart, genital herpes, chancroid, hepatitis B, chlamydia, gonorrhea, non-gonococcal and non-chlamydial urethritis, mpox and proctitis.

After the interview, blood samples were collected for STIs screening (HIV, syphilis, hepatitis B and C), using rapid immunochromatographic tests, as recommended by the Brazilian Ministry of Health [17]. Serum samples were tested for IgM and total anti-HAV antibodies by a commercial chemiluminescent immunoassay kit (Diasorin LIAISON^®^ Anti-HAV and HAV IgM, Italy) in the Central Public Health Laboratory of Rio Grande do Norte (LACEN/RN) [24]. The sample collection, transport and storage followed the standard procedures from LACEN/RN, as well as the validation process and quality control of laboratory tests.

A descriptive analysis was conducted initially through the presentation of categorical variables by absolute and relative values and continuous variables with non-normal distribution by median (interquartile range). The prevalence of HAV susceptibility was calculated with 95% confidence interval (95% CI). The HAV immune status (susceptibility/immunity) was the dependent variable and the independent variables were the sociodemographic characteristics and clinical information. Missing data occurred in only two independent variables in “sexual practices” category by lack of response and represented at most 1% of the data. Therefore, missing data were excluded from analysis. Bivariate analyses were performed with Pearson chi-square or Fisher’s exact tests for categorical variables. The linear-by-linear association chi-square test was used for ordinal variables. To assess the factors associated with HAV susceptibility, the magnitude of association was verified by Prevalence Ratio (PR) and respective 95% CI.

Independent variables with p-value <0.20 in bivariate analysis were considered for multivariate Poisson regression analysis, with a robust variance. To maintain the variables in multivariate analysis, multicollinearity tests were performed. Multicollinearity was measured by crossing the independent variables, and those with p-values <0.001 were considered collinear. From this, the variables presenting the best correlation with the theoretical model were chosen. The objective of the robust Poisson regression model was to check the individual effect of each independent variable remaining in the model on HAV susceptibility, calculating the adjusted PR and respective 95% CI. All reported values are two-tailed. A p-value <0.05 was considered statistically significant. The data were analyzed using SPSS package (IBM Corp. Released 2011. IBM^®^ SPSS^®^ Statistics for Windows^®^, Version 20.0. Armonk, NY: IBM Corp.).

## Results

### Study population characteristics

Between September 2021 and June 2023, 310 individuals were potentially eligible and invited to participate in the study. Two hundred and eighty-two (282/310; 90.9%) agreed to participate in the study and all of them answered the questionnaire and provided a blood sample to perform the STI screening and anti-HAV antibodies testing. Table 1 displays details about the sociodemographic profile of the study population.

**Table 1:** Sociodemographic profile of cisgender MSM taking HIV PrEP from specialized care service of State of Rio Grande do Norte, in Northeastern Brazil, between 2021 and 2023.

	Total	
Variable	N = 282	%
**Age (years)**		
18–24	29	10.3
25–29	87	30.9
30–39	121	42.9
40–49	36	12.8
≥ 50	9	3.2
**Race/Skin color (self-declared)**		
White	126	44.7
Black	39	13.8
Brown	114	40.4
Indigenous	3	1.1
**Marital status**		
Single/ Divorced	233	82.6
Civil Union/ Married	49	17.4
**Sexual orientation (self-declared)**		
Gay/ homosexual	222	78.7
Bisexual	60	21.3
**Length of schooling (years)**		
≤ 11	35	12.4
> 11	247	87.6
**Income (minimum wages)** ^a^		
< 2.0	81	28.7
2.0–5.0	143	50.7
> 5.0	58	20.6
**Health insurance**		
Yes	147	52.1
No	135	47.9

MSM: men who have sex with men; HIV: human immunodeficiency virus; PrEP: pre-exposure prophylaxis.

^a^ One minimum wage represented approximately R$ 1,204.00 (U$ 247.64 USD).

### Vaccination status for hepatitis, sexual and non-sexual behavioral characteristics and medical history of sexually transmitted infections

Most of participants (75.9%) received the full hepatitis B vaccine schedule. In turn, only 12.1% received at least one dose of hepatitis A vaccine. The majority of PrEP users engaged in oral sex (95.4%) and condomless anal intercourse (83.7%), reported low frequency of condom use (54.2%) and had ≥3 partners (59.1%), having sex only with cisgender MSM in 90.9% in the past 3 months. Fifty-three percent had a steady partner. Most of individuals reported practice of rimming (92.6%), group sex (73.5%) and fingering (59.6%). About two thirds of subjects reported use of geosocial dating app, binge drinking and use of HIV PrEP ≥6 months. A lifetime history of STIs was reported by 64.2% and the most common STIs were syphilis (63.0%), urethritis (35.9%) and hepatitis B (15.4%). Table 2 provides details on vaccination status, sexual and non-sexual behaviors and medical history of STI.

**Table 2:** Profile of vaccination status for hepatitis, behavioral characteristics and medical history of STIs among cisgender MSM taking HIV PrEP from specialized care service of State of Rio Grande do Norte, in Northeastern Brazil, between 2021 and 2023.

	Total	
Variable	N = 282	%
**Complete hepatitis B vaccine schedule**		
No	68	24.1
Yes	214	75.9
**Hepatitis A vaccine (at least one dose)**		
No	248	87.9
Yes	34	12.1
**Condomless anal intercourse in the past 3 months** ^a^		
No	43	16.3
Yes	220	83.7
**Sexual role during condomless anal intercourse in the past 3 months** ^a^		
Anal sex with condom (*Activo*, *Pasivo* or *Moderno*)	43	15.2
Only receptive (*Pasivo*)	39	17.7
Receptive and insertive (Versatile or *Moderno*)	101	45.9
Only insertive (*Activo*)	80	36.4
**Frequency of condom use during intercourse in the past 3 months** ^b^		
Never once	63	23.9
Less than half of the time	46	17.4
Half of the time	34	12.9
More than half of the time	78	29.5
Everytime	43	16.3
**Number of sexual partners in the past 3 months** ^b^		
≤ 2	108	40.9
3–5	74	28.0
> 5	82	31.1
**Gender of sexual partners in the past 3 months** ^b^		
Only cisgender men partners	240	90.9
Cis and transgender men and women partners and nonbinary people	24	9.1
**Steady partner**		
No	131	46.5
Yes	151	53.5
**Exchange sex for money, housing, drugs and/or goods (ever)**		
No	245	86.9
Yes	37	13.1
**Sex worker**		
No	275	97.5
Yes	7	2.5
**Oral sex in the past 3 months**		
No	13	4.6
Yes	269	95.4
**Sexualized drug use in the past 3 months** ^c^		
No	229	82.1
Yes	50	17.9
**Gay sauna attendance for sexual encounter (ever)**		
No	171	60.6
Yes	111	39.4
**Sharing insertive sex toys with partners during intercourse (ever)**		
No	218	77.3
Yes	64	22.7
**Fisting (ever)**		
No	225	79.8
Yes	57	20.2
**Anal fingering (ever)**		
No	114	40.4
Only receptive	16	5.7
Receptive and insertive	102	36.2
Only insertive	50	17.7
**Rimming (ever)**		
No	21	7.4
Only receptive	27	9.6
Receptive and insertive	195	69.1
Only insertive	39	13.8
**Group sex (ever)** ^d^		
No	74	26.5
Yes	205	73.5
**Binge drinking in the past 3 months**		
No	102	36.2
Yes	180	63.8
**Substance use in the past 3 months**		
No	179	63.5
Yes	103	36.5
**Erectile dysfunction drugs use in the past 3 months**		
No	256	90.8
Yes	26	9.2
**Use of geosocial dating app in the past 3 months**		
No	92	32.6
Yes	190	67.4
**Length of HIV PrEP use**		
Starting HIV PrEP	99	35.1
6 months of HIV PrEP	84	29.8
≥ 12 months of HIV PrEP	99	35.1
**Any sexually transmitted infection in the past 6 months**		
No	225	79.8
Yes	57	20.2
**Any lifetime sexually transmitted infection**		
No	101	35.8
Yes	181	64.2

STIs: sexually transmitted infections; MSM: men who have sex with men; HIV: human immunodeficiency virus; PrEP: pre-exposure prophylaxis.

^a^ Total number of participants: 263.

^b^ Total number of participants: 264.

^c^ Total number of participants: 280.

^d^ Total number of participants: 279.

### Prevalence and factors associated with HAV susceptibility

Reactive total anti-HAV antibodies were interpreted as immunity to HAV due to past HAV infection or vaccination, while non-reactive results were interpreted as HAV susceptibility. None of the samples were reactive for IgM anti-HAV antibody. There were no borderline results. Non-reactive results of total anti-HAV antibodies were found in 106 individuals (37.6%; 95% CI 32.1–43.3) of the population. In bivariate analysis, HAV susceptibility was associated with age <30 years, being white, unmarried, using condom more than half of time or all of time, intercourse only with cisgender men, non-steady partner, not attending gay sauna for sex, practice of fingering (more strongly with only receptive fingering) and no lifetime history of STIs. There was a significant association between HAV susceptibility and increasing use of condom (p = 0.020). In multivariate analysis, the following variables remained significant: age <30 years (PR: 2.02; 95% CI 1.61–2.53; p<0.0001), having health insurance (PR: 1.39; 95% CI 1.19–1.64; p<0.001), having sex only with cisgender men (PR: 1.52; 95% CI 1.23–1.89; p< 0.001), non-steady partner (PR: 1.20; 95% CI 1.01–1.43; p = 0.042) and no lifetime history of STIs (PR: 1.25; 95% CI 1.03–1.53; p = 0.026). Tables 3 and 4 show the sociodemographic and clinical characteristics and sexual behaviors associated with HAV susceptibility.

**Table 3:** Sociodemographic and clinical factors associated with susceptibility to HAV (non-reactive total anti-HAV antibodies) among cisgender MSM taking HIV PrEP from specialized care service of State of Rio Grande do Norte, in Northeastern Brazil, between 2021 and 2023.

	Total anti-HAV antibodies		Bivariate analysis			Multivariate analysis ^a^	
Variable	Non-reactive N (%)	Reactive N (%)	PR (95% CI)	P-value	Cramer’s V	PR (95% CI)	P-value
**Age (years)**							
< 30	69 (59.5)	47 (40.5)	2.67 (1.94–3.68)	<0.001^b^	0.378	2.02 (1.61–2.53)	<0.001
≥ 30	37 (22.3)	129 (77.7)					
**Race/ Skin color (self-declared)**							
White	59 (46.8)	67 (53.2)	1.52 (1.13–2.06)	0.006^b^	0.165		
Black	47 (30.7)	106 (69.3)					
**Marital status**							
Single/ Divorced	96 (41.2)	137 (58.8)	2.02 (1.14–3.58)	0.006^b^	0.163		
Civil union/ Married	10 (20.4)	39 (79.6)					
**Sexual orientation (self-declared)**							
Gay/homosexual	88 (39.6)	134 (60.4)	1.32 (0.87–2.01)	0.171^b^	0.081		
Bisexual	18 (30.0)	42 (70.0)					
**Length of schooling (years)**							
> 11	96 (38.9)	151 (61.1)	1.36 (0.79–2.35)	0.239^b^	0.070		
≤ 11	10 (28.6)	25 (71.4)					
**Monthly income (national minimum wage)** ^c^							
< 2.0	29 (35.8)	52 (64.2)	1.00	0.227^b^	0.103		
2.0–5.0	60 (42.0)	83 (58.0)	1.17 (0.83–1.66)				
> 5.0	17 (29.3)	41 (70.7)	0.82 (0.50–1.34)				
**Health insurance**							
Yes	62 (42.2)	85 (57.8)	1.29 (0.95–1.76)	0.097^b^	0.099	1.39 (1.19–1.64)	< 0.001
No	44 (32.6)	91 (67.4)					
**Complete hepatitis B vaccine schedule**							
No	24 (35.3)	44 (64.7)	0.92 (0.64–1.33)	0.654^b^	0.027		
Yes	82 (38.3)	132 (61.7)					
**Hepatitis A vaccine (at least one dose)**							
No	96 (38.7)	152 (61.3)	1.32 (0.76–2.27)	0.294^b^	0.063		
Yes	10 (29.4)	24 (70.6)					
**Binge drinking in the past 3 months**							
No	34 (33.3)	68 (66.7)	0.83 (0.60–1.16)	0.267^b^	0.066		
Yes	72 (40.0)	108 (60.0)					
**Substance use in the past 3 months**							
No	60 (33.5)	119 (66.5)	0.75 (0.56–1.01)	0.063^b^	0.111		
Yes	46 (44.7)	57 (55.3)					
**Erectile dysfunction drug use in the past 3 months**							
No	98 (38.3)	158 (61.7)	1.24 (0.69–2.26)	0.451^b^	0.045		
Yes	8 (30.8)	18 (69.2)					
**Geosocial dating app use in the past 3 months**							
No	31 (33.7)	61 (66.3)	0.85 (0.61–1.20)	0.348^b^	0.056		
Yes	75 (39.5)	115 (60.5)					
**Length of HIV PrEP use**							
Starting HIV PrEP	43 (43.4)	56 (56.6)	1.26 (0.93–1.71)	0.136^b^	0.089		
Six months or more	63 (34.4)	120 (65.6)					
**Any STI in the past 6 months**							
No	89 (39.6)	136 (60.4)	1.33 (0.86–2.04)	0.175^b^	0.081		
Yes	17 (29.8)	40 (70.2)					
**Any lifetime STI**							
No	46 (45.5)	55 (54.5)	1.37 (1.02–1.85)	0.039^b^	0.123	1.25 (1.03–1.53)	0.026
Yes	60 (33.1)	121 (66.9)					

HAV: hepatitis A virus; MSM: men who sex with men; HIV: human immunodeficiency virus; PrEP: pre-exposure prophylaxis; PR: prevalence ratio; 95% CI: 95% confidence interval; STI: sexually transmitted infection.

^a^ Model adjusted for the variables: race/skin color (self-declared), fingering (ever), length of HIV PrEP use and substance use in the past 3 months.

^b^ Pearson’s chi—square test.

^c^ One minimum wage represented approximately R$ 1,204.00 (U$ 247.64 USD).

**Table 4:** Sexual behaviors associated with susceptibility to HAV (non-reactive total anti-HAV antibodies) among cisgender MSM taking HIV PrEP from specialized care service of State of Rio Grande do Norte, in Northeastern Brazil, between 2021 and 2023.

	Total anti-HAV antibodies		Bivariate analysis			Multivariate analysis ^a^	
Variable	Non-reactive N (%)	Reactive N (%)	PR (95% CI)	P-value	Cramer’s V	PR (95% CI)	P-value
**Condomless anal intercourse in the past 3 months**							
No	17 (39.5)	26 (60.5)	1.09 (0.72–1.64)	0.693^b^	0.024		
Yes	80 (36.4)	140 (63.6)					
**Sexual role during condomless anal intercourse in the past 3 months**							
Anal sex with condom (*Activo*, *Pasivo* or *Moderno*)	17 (39.5)	26 (60.5)	1.00	0.895^b^	0.048		
Only receptive (*Pasivo*)	16 (41.0)	23 (59.0)	1.04 (0.61–1.76)				
Receptive and insertive (Versatile or *Moderno*)	36 (35.6)	65 (64.4)	0.90 (0.57–1.42)				
Only insertive (*Activo*)	28 (35.0)	52 (65.0)	0.89 (0.55–1.42)				
**Frequency of condom use during intercourse in the past 3 months**							
Never once	16 (25.4)	47 (74.6)	1.00	0.063^b^	0.145		
Less than half—half of the time	29 (36.2)	51 (63.7)	1,43 (0,85–2,39)				
More than half—everytime	52 (43.0)	69 (57.0)	1,69 (1,06–2,71)				
**Number of sexual partners in the past 3 months**							
≤ 2	38 (35.2)	70 (64.8)	1.11 (0.74–1.67)	0.226^b^	0.106		
3–5	33 (44.6)	41 (55.4)	1.41 (0.94–2.11)				
> 5	26 (31.7)	56 (68.3)	1.00				
**Gender of sexual partners in the past 3 months**							
Only cisgender men	95 (39.6)	145 (60.4)	4.75 (1.25–18.07)	0.002^b^	0.186	1.52 (1.23–1.89)	<0.001
Cis and transgender men and women and nonbinary people	2 (8.3)	22 (91.7)					
**Steady sexual partner**							
No	59 (45.0)	72 (55.0)	1.45 (1.07–1.96)	0.016^b^	0.143	1.20 (1.01–1.43)	0.042
Yes	47 (31.1)	104 (68.9)					
**Exchange sex for money, housing, drugs and/or goods (ever)**							
No	93 (38.0)	152 (62.0)	1.08 (0.68–1.72)	0.741^b^	0.020		
Yes	13 (35.1)	24 (64.9)					
**Sex worker**							
No	104 (37.8)	171 (62.2)	1.32 (0.41–4.31)	0.715^c^	0.030		
Yes	2 (28.6)	5 (71.4)					
**Oral sex in the past 3 months**							
No	5 (38.5)	8 (61.5)	1.02 (0.51–2.07)	1.000^c^	0.004		
Yes	101 (37.5)	168 (62.5)					
**Sexualized drug use in the past 3 months**							
No	89 (38.9)	140 (61.1)	1.14 (0.75–1.74)	0.521^b^	0.038		
Yes	17 (34.0)	33 (66.0)					
**Gay sauna attendance (ever)**							
No	73 (42.7)	98 (57.3)	1.44 (1.03–2.01)	0.028^b^	0.131		
Yes	33 (29.7)	78 (70.3)					
**Sharing insertive sex toys with partners during intercourse (ever)**							
No	81 (37.2)	137 (62.8)	0.95 (0.67–1.35)	0.782^b^	0.016		
Yes	25 (39.1)	39 (60.9)					
**Fisting (ever)**							
No	88 (39.1)	137 (60.9)	1.24 (0.82–1.88)	0.294^b^	0.062		
Yes	18 (31.6)	39 (68.4)					
**Anal fingering (ever)**							
No	28 (24.6)	86 (75.4)	1.00	<0.001^b^	0.281		
Only receptive	11 (68.8)	5 (31.2)	2.80 (1.77–4.44)				
Receptive and insertive	51 (50.0)	51 (50.0)	2.04 (1.40–2.96)				
Only insertive	16 (32.0)	34 (68.0)	0.78 (0.45–1.34)				
**Rimming (ever)**							
No	4 (19.0)	17 (81.0)	1.00	0.264^b^	0.119		
Only receptive	11 (40.7)	16 (59.3)	2.14 (0.79–5.77)				
Receptive and insertive	78 (40.0)	117 (60.0)	2.10 (0.86–5.16)				
Only insertive	13 (33.3)	26 (66.7)	1.75 (0.65–4.70)				
**Group sex (ever)**							
No	29 (39.2)	45 (60.8)	1.04 (0.75–1.46)	0.805^b^	0.015		
Yes	77 (37.6)	128 (62.4)					

HAV: hepatitis A virus; MSM: men who sex with men; HIV: human immunodeficiency virus; PrEP: pre-exposure prophylaxis; PR: prevalence ratio; 95% CI: 95% confidence interval.

^a^ Model adjusted for the variables: race/skin color (self-declared), fingering (ever), length of HIV PrEP use and substance use in the past 3 months.

^b^ Pearson’s chi—square test.

^c^ Fisher’s exact test.

## Discussion

We identified a high proportion of cisgender MSM that are HAV susceptible at a public HIV PrEP service in a capital state of Northeastern Brazil. In this study, we found that age, socioeconomic status and sexual characteristics, such as partnerships and medical history of STI, were associated with HAV susceptibility. Data on status of immunity to HAV in MSM are scarce in Brazil, hindering the development of public policies focused on prevention.

The observed frequency of HAV susceptibility (37.6%; 95% IC: 32.1–43.3) in study participants was lower than that detected among MSM population samples from the Netherlands (63.0%; 95% IC: 60.0–65.0) [18], Estonia (68.0%), [25], France (50.4%), [26] and Italy (57.2%), [27] but similar to rates found in USA (42.0%), [28] Australia (32.0%; 95% CI 29.0–35.0), [29] and United Kingdom (45%; 95% CI: 42.0–47.0) [30] and very similar to that found in a study with MSM from Central Brazil (37.7%) [19]. These regional differences may be explained mainly by distinct socioeconomic patterns among the countries, that are inversely correlated with HAV endemicity levels, but also may result from varying degrees of HAV exposure through sexual behaviors [2, 4].

When the prevalence of HAV susceptibility is >30% in MSM communities, there is a risk of future outbreaks due to sustained viral transmission through sexual contact. But the appropriate level of immunity can be achieved through vaccination [20]. For adults, the decision to test before vaccination should consider: the expected prevalence of immunity in population and the cost of vaccination compared with that of serologic testing [31]. In our context, given the HAV seroprevalence in MSM population is > 33% and the cost of screening for hepatitis A (including serologic testing and laboratory/office visits) is less than one third the cost of the vaccination series, testing MSM for total Anti-HAV antibodies and then vaccinating those susceptible is more cost-effective than vaccinating everyone [15, 32, 33].

Only approximately 10% of participants self-reported receipt of at least one dose of the hepatitis A vaccine. This is because Brazilian public policy for adult vaccination against hepatitis A does not include MSM [7]. However, in 2018, the Brazilian Ministry of Health expanded the indication of the hepatitis A vaccine to MSM, but only for São Paulo State due to the 2017–2018 hepatitis A outbreak [32]. Even in regions where hepatitis A vaccine is routinely recommended for MSM, the vaccination rates remain low, ranging from 40.3% to 48.0% [28, 34]. The reasons behind the low coverage are unclear, but may be explained by the lack of healthcare provider knowledge about hepatitis A vaccine indications [28]. Another reason may be, in our context, the vaccine costs in private clinics.

Inversely, three quarters of participants self-reported full hepatitis B vaccination. The complete hepatitis B vaccination was significantly associated with protection against hepatitis B virus, defined by hepatitis B surface antibody (anti-HBs) concentration >10mIU/mL (PR: 2.11; 95% CI: 1.53–2.91, p<0.0001; Cramer’s V: 0.424) (S1 Table). There was no association between hepatitis A and B vaccination (PR: 1.14; 95% CI: 0.98–1.34, p = 0.171; Cramer’s V: 0.081) (S1 Table). This is justified by the fact that the hepatitis B vaccine is available free of charge to all unvaccinated Brazilian citizens, regardless of age.

A high proportion of cisgender MSM of the study reported binge drinking and substance abuse, use of geosocial dating apps, multiple sexual partners, condomless anal intercourse, fingering, rimming and group sex, putting them at risk of STIs acquisition/transmission. Although no association was established with HAV immune status in this study, these sexual practices have been implicated as the transmission route in hepatitis A outbreaks affecting MSM [6–12].

The use of HIV PrEP among MSM is increasing and is associated with a greater frequency of condomless anal intercourse, condomless sex with an HIV-positive or HIV-unknown partner, number of sexual partners and diagnosis of any STI [35]. However, PrEP services offer opportunity to improve the STI control and prevention, including HAV infection. In regions where free-of-charge HAV vaccination is not part of overall PrEP care, as Brazil, the healthcare providers should screen patients for HAV, recommend and encourage hepatitis A vaccination for susceptible people and educate them about modes of transmission, symptoms and signs and preventive measures. Besides, unvaccinated MSM should be monitored for the disease on a regular basis during the follow-up [13, 17, 28].

Young age and having health insurance, as a proxy for better socioeconomic status, were significantly associated with HAV susceptibility in this study, consistent with results reported by other authors [18, 19, 26, 27, 29, 30]. Fecal-oral transmission of HAV is associated with lack of access to safe water and sanitation facilities and poor hygiene [3, 36]. As socioeconomic, hygienic and sanitation conditions, the HAV infection rate declines and people become infected at a later age [36]. This is paradoxically problematic, because the morbidity and severity of HAV infection increase with age.

Sixty-four percent of study participants reported any STI throughout the life. We observed a significantly higher proportion of HAV susceptible subjects without medical history of STI than those with a lifetime history of STI. A greater burden of STIs are reported among PrEP-using MSM, when compared with those not taking prophylaxis and general population [35, 37, 38]. Our results are in trends with previous studies [18, 27, 29]. This association between traditional STIs and HAV infection can be inferred by the fact that HAV and other sexual pathogens share some overlapping behaviors and transmission routes.

We found two partnership-level factors associated with HAV immune status: gender identity of sexual partners and having a non-steady partner. Participants who had sex only with cisgender men were proportionally more HAV susceptible than those having sex with cisgender, transgender and/or nonbinary people. Less repertoires of sexual partnerships may mean less exposure to sexually transmitted pathogens, including HAV. The need and desire of affirming the gender identity may lead trans people to engage in anonymous and condomless sex more frequently when compared to cis people [39, 40]. A previous study also demonstrated that the incidence of HIV, other STIs and viral hepatitis varied significantly among the different categories of gender identity [41].

Having a non-steady sexual partner was statistically associated with HAV susceptibility. Non-stable partnerships include casual and anonymous sexual relations and they usually do not carry great levels of familiarity, commitment and/or intimacy. Hence, individuals having non-steady partners are more likely to engage to use condom during anal intercourse [42]. We observed a significantly increasing condom use during intercourse among participants having a non-steady partner (p<0.001), reinforcing this hypothesis (S2 Table).

This study has some limitations. Some factors potentially associated with HAV transmission were not addressed, such as international travel, crowding at home, family size, housing condition, access to water and sanitation facilities. However, in regions with moderate/high water access rates, as in Brazil, socioeconomic indicators seem to be better predictors of HAV exposure than water and sanitation variables, as we explored [3]. We may not have observed an association between sexual practices and HAV immune status, because we investigated mostly sexual behaviors in the previous three months. Lifetime sexual behaviors could be more appropriate sexual markers for HAV exposure. The medical history of STI indirectly provided information on sexual health of participants throughout life and we detected its association with HAV susceptibility in the present work. The non-random selection of convenience sampling method may have recruitment bias. Furthermore, PrEP services, as well as STI clinics, may attract people who perceive themselves to be at high risk of STIs. Both issues may undermine the representation of overall cisgender MSM population.

In spite of these limitations, this study is the first research about HAV susceptibility among cisgender MSM receiving HIV PrEP in Northeastern Brazil. The population sample corresponded to approximately 40% of all PrEP users in the state. HIV PrEP has been known to change sexual and non-sexual behaviors among their users. Although it may not be extrapolated to all MSM population, this study contributed to outline the prevalence and factors associated with risk for hepatitis A in this subgroup. Understanding the epidemiology of HAV infection in key populations is essential to development and implementation of targeted preventive strategies, especially following the recent HAV outbreak in Brazil.

## Conclusions

A high proportion of MSM taking HIV PrEP were HAV susceptible, putting them at risk for sustained transmission and future outbreaks of hepatitis A. Sexual practices that enable fecal-oral contact have been implicated in HAV transmission among MSM, although they were not associated with immune status of HAV in this work. HAV susceptibility was associated with young age, better socioeconomic status, no history of STIs, having a non-steady partner and intercourse only with cisgender men. Knowing the proportion of MSM susceptible to HAV and identifying the factors associated with HAV susceptibility can support the formulation and implementation of public policies aimed at preventing the disease in key populations, such as MSM.

## Supporting information

S1 Table Analysis of factors associated with hepatitis B vaccination. (DOCX)

S2 Table Analysis of association of condom use with non-steady partner. (DOCX)
