# Supplementary material for: Factors associated with hepatitis A susceptibility among men who have sex with men using HIV pre-exposure prophylaxis in Northeastern Brazil: A cross-sectional study
Source: PLoS One. 2024 Mar 28;19(3):e0301397. doi: 10.1371/journal.pone.0301397 (PMC10977755; doi:10.1371/journal.pone.0301397)
Supplement: S1 Table — (DOCX) [file pone.0301397.s001.docx]

S1 Table. Analysis of factors associated with hepatitis B vaccination among cisgender MSM taking HIV PrEP from specialized care service of Rio Grande do Norte, in Northeastern Brazil, between 2021 and 2023.

| **Variable** | **Complete hepatitis B vaccine schedule**  **(Self-report)** | | **Crude**  **PR** | **95% CI** | **p** | **Cramer’s V** |
| --- | --- | --- | --- | --- | --- | --- |
|  | **Yes** | **No** |  |  |  |  |
|  | **N (%)** | **N (%)** |  |  |  |  |
| **Anti-HBs^a^** |  |  |  |  |  |  |
| Reactive | 191 (85.3) | 33 (14.7) | 2.11 | (1.53 – 2.91) | < 0.001^b^ | 0.424 |
| Non-reactive | 23 (40.4) | 34 (59.6) |  |  |  |  |
| **Hepatitis A vaccine (at least one dose) – self-report** |  |  |  |  |  |  |
| Yes | 29 (85.3) | 5 (14.7) | 1.14 | (0.98 – 1.34) | 0.1.71^b^ | 0.081 |
| No | 185 (74.6) | 63 (25.4) |  |  |  |  |

MSM: men who sex with men. HIV: human immunodeficiency virus. PrEP: pre-exposure prophylaxis. PR: prevalence ratio. 95% CI: 95% confidence interval. Anti-HBs: hepatitis B surface antibody.

^a^ Reactive Anti-HBs is defined by concentration >10mIU/mL. Non-reactive result is defined by concentration ≤ 10mIU/mL.

^b^ Pearson’s chi – square test.
