# Supplementary material for: Factors associated with hepatitis A susceptibility among men who have sex with men using HIV pre-exposure prophylaxis in Northeastern Brazil: A cross-sectional study
Source: PLoS One. 2024 Mar 28;19(3):e0301397. doi: 10.1371/journal.pone.0301397 (PMC10977755; doi:10.1371/journal.pone.0301397)
Supplement: S2 Table — (DOCX) [file pone.0301397.s002.docx]

S2 Table. Analysis of association of condom use with steady partner among cisgender MSM taking HIV PrEP from specialized care service of Rio Grande do Norte, in Northeastern Brazil, between 2021 and 2023.

| **Variable** | **Steady partner** | | **Crude**  **PR** | **95% CI** | **p** | **Cramer’s V** |
| --- | --- | --- | --- | --- | --- | --- |
|  | **No** | **Yes** |  |  |  |  |
|  | **N (%)** | **N (%)** |  |  |  |  |
| **Frequency of condom use during intercourse in the past 3 months** |  |  |  |  |  |  |
| Never once | 13 (20.6) | 50 (79.4) | 1 | - | < 0.001^a,b^ | 0.270 |
| Less than half – half of the time | 39 (48.8) | 41 (51.2) | 2.36 | (1.39 – 4.03) |  |  |
| More than half – everytime | 65 (53.7) | 56 (46.3) | 2.60 | (1.56 – 4.34) |  |  |

MSM: men who sex with men. HIV: human immunodeficiency virus. PrEP: pre-exposure prophylaxis. PR: prevalence ratio. 95% CI: 95% confidence interval.

^a^ Pearson’s chi – square test.

^b^ Linear-by-linear association chi-square test.
